# Prevalence of Three-Rooted Deciduous Mandibular Molars in the Children of Northwestern Iran

**DOI:** 10.1155/2021/5643668

**Published:** 2021-10-26

**Authors:** Maryam Khosrozadeh, Maryam Mostafavi, Mohammad Hassan Hamrah, Elham Niknejad

**Affiliations:** ^1^Department of Pediatric Dentistry, School of Dentistry, Urmia University of Medical Sciences, Urmia, Iran; ^2^Department of Oral and Maxillofacial Radiology, School of Dentistry, Urmia University of Medical Sciences, Urmia, Iran; ^3^Department of Pediatric Dentistry, School of Dentistry, Tehran University of Medical Sciences, Tehran, Iran

## Abstract

**Introduction:**

Although primary teeth have a determinative role in development of normal occlusion, few studies about anomalies related to deciduous dentition have been conducted so far. Regarding recent improvements in common knowledge and the importance of maintaining primary teeth until eruption of succedaneous teeth, identifying the morphology of primary teeth and probable variations is of great importance to achieve optimal therapeutic outcome. This study aims to determine the prevalence of three-rooted mandibular primary molars in a population of northwestern Iran.

**Materials and Methods:**

In this descriptive cross-sectional study, periapical radiographs of patients attending private oral and maxillofacial radiology offices in the northwestern region of Iran from 2017 to 2019 were retrospectively reviewed. A total of 300 cases in the 3–10-year age range having bilateral periapical radiographs from mandibular primary molars were screened. First and second primary molars were observed meticulously. Gender, side of the mandible (right or left), symmetry, overall prevalence, and prevalence considering the type of molar (D or E) were recorded and analyzed.

**Results:**

Overall prevalence of three-rooted mandibular primary molars was 9.33% ( 28/300), 92.9% of which were unilateral. The prevalence of teeth showing supernumerary roots among all teeth examined was 2.5% (30/1200). Considering symmetry and gender, the occurrence of these three-rooted primary molars did not differ significantly (respectively, *p*=0.832 and *p*=0.541). However, there was a significant relationship between the occurrence of supernumerary roots and left side for three-rooted first molars and right side for three-rooted second molars (*p*=0.021).

**Conclusion:**

Obtained data show that three-rooted mandibular primary molars in children of northwest region of Iran have a prevalence of approximately 10 % which urges some specific considerations in exodontic and endodontic procedures.

## 1. Introduction

An understanding of root and canal morphology in the human dentition is necessary since a lack of understanding is often associated with inability to properly locate, instrument, irrigate, and fill canals during root canal treatment [1–3].

Primary molars erupt between 21 and 33 months of age. In cases where there is a long time left until the eruption of succedaneous tooth, we can prevent tooth extraction and further orthodontic problems by debridement of the root canals of the deciduous tooth and maintaining nonpathological conditions until exfoliation [4–6].

Newly erupted primary mandibular molars represent a simple root canal system including one canal in each of the mesial and distal roots. Secondary dentin deposition, canal narrowing, and the physiological resorption process all contribute to the final complexity of root canal system [7].

As functional components, primary teeth have a determining role in the health and development of the child. They are also responsible for optimal mastication and digestion, phonation, swallowing, stimulating proper growth of the jaw, and maintaining the space required for the eruption of permanent teeth [8, 9].

As a result of complicated molecular interactions in the way to form a tooth, everything may not go as planned [10]. Concrescence, dilacerations, and hypercementosis are the most common root anomalies in primary dentition [11]. Carabelli was the first to describe the existence of an extra or supernumerary root in permanent mandibular molars which he termed “Radix Entomolaris” [12]. This extra root is usually smaller than the distal root, and its specific curvature urges extra attention during root canal therapy. Many cases of three-rooted molars in permanent dentition have been recorded since then [11, 13–15].

While the etiology of supernumerary roots in permanent dentition is unknown, factors such as external environmental stimuli during odontogenesis, greater penetrance of the atavistic gene, and ethnic and genetic influences are believed to be the main causes. The same can most likely be attributed to the deciduous dentition [16].

Considered to be a racial characteristic of certain Indian, Asian, Arctic, and North American populations, three-rooted mandibular molars appear to be more common in permanent dentition and have a frequency of less than 1% in primary dentition according to Tratman [17, 18].

Despite numerous studies regarding this issue in permanent teeth, only a few studies have focused on primary molars and the majority of them are case studies [19].

In a study of 500 extracted first and second permanent molars of the mandible from Iranian population, Kuzekanani et al. reported that the prevalence of Radix Entomolaris (RE) was 6% in the first and 0.8% in the second molars [20].

Rahimi et al. also reported a prevalence of 3% after investigating 386 CBCTs related to the first permanent mandibular molars of Iranian patients and obtaining reconstructed axial images of these teeth, which was slightly higher in women [21].

Given the known ethnic variations in supernumerary roots and the lack of data on contemporary populations in the Middle East, the present study followed a cross-sectional design to assess the occurrence of three-rooted primary mandibular molars in the Iranian population, based on radiographic examination of 300 patients with bilateral periapical radiographs.

## 2. Materials and Methods

A total of 300 intraoral periapical radiographs of bilateral primary mandibular molars, from January 2017 to June 2019, were collected for retrospective screening and examination.

These images were prescribed by pediatric dentists for clinical examinations and pulp treatments and were obtained in private specialized offices of northwestern Iran under the supervision of oral and maxillofacial radiologists.

To reduce error and obtain adequate images, intraoral periapical radiographs were taken using the standardized paralleling cone technique with the aid of X-ray film positioning device, Rinn XCP (Rinn Corporation, Elgin, IL, USA).

The target age group was in the range of three to ten years consisting of 165 girls and 135 boys. Blurry or inaccurate radiographs or images with artifacts were excluded to minimize radiographic misinterpretation. We also eliminated images showing oligodontia or tooth extraction, radiolucency or furcation involvement, tooth malposition, physiologic root resorption due to the child's age or pathologic resorption as a result of periapical infection affecting more than 1/3 of the root, and molars with incomplete root formation less than 2/3 of a normal primary mandibular molar root.

The ethical clearance for this study was obtained from the ethics committee of Urmia University of Medical Sciences (IR.UMSU.REC.1398.20).

A qualified pediatric dentist and an oral and maxillofacial radiologist examined the digital periapical images in a calibrated screen. In this investigation, an extra root was defined as a clearly distinguishable independent extra root, evident as the crossing of translucent lines defining the pulp and periodontal ligament spaces [22, 23]. Regardless of the location, any extra root with the aforementioned criteria was considered an additional root.

Disagreements in the interpretation of radiographs were resolved by consensus between the two investigators. To determine interexaminer reliability, the kappa value was calculated. The prevalence of bilateral and unilateral, right- or left-side three-rooted deciduous mandibular molars as well as their gender predilection was studied.

Central and dispersion indices (mean and standard deviation) were used to describe quantitative data, and prevalence and prevalence percentage were used for qualitative data. To make comparisons, we used appropriate statistical tests as needed, such as chi-square test and *t*-test. All statistical analyses were performed using SPSS-21 software (IBM Corp., Armonk, NY, USA) with significance set at *p* < 0.05.

## 3. Results

Due to the fact that the diagnosis was affected by the accuracy and expertise of the individual, 30 samples were randomly reviewed by a blinded qualified observer and Cohen's kappa coefficient was calculated to measure interrater reliability. Based on the results, to determine the type of tooth with extra roots, the kappa value was reported to be 1, which shows a very good agreement. The value was calculated to be 0.76 for first and 0.783 for second primary molars, which shows a good and acceptable accordance.

Out of a total of 300 children studied, the gender distribution of patients consisted of 65% girls and 35% boys and 28 cases had three-rooted primary mandibular molars. The overall prevalence was calculated to be 9.33%. The prevalence of mandibular three-rooted deciduous molars among the total number of examined teeth was 2.5% (30/1200). Of these, 16 cases (57.1%) were related to first and 12 cases (42.8%) were related to second primary molars.

Extra root was observed in 15 girls (57.53%) and 13 boys (42.46%). The chi-square test was used to compare the sex distribution according to the type of tooth with three roots. The results showed that there was no statistically significant difference based on the sex distribution between the groups (*p*=0.541; Table 1).

From the perspective of the side of the mandible where three-rooted teeth were detected, by type of deciduous molars, among the whole three-rooted first primary molars, 68.8% (11 cases) of them were on the left, 25% (4 cases) on the right, and 6.2% (1 case) were bilateral. The distribution pattern of the three-rooted second primary molars was 16.7% left, 75% right, and 8.3% bilateral. The chi-square test was used to compare the distribution of three-rooted teeth according to the mandibular side, which showed that there was a statistically significant difference based on the side of the mandible between the groups (*p*=0.021; Table 2).

According to the findings, out of the total teeth with supernumerary roots, 26 cases (92.9%) were unilateral and 2 cases (7.1%) were bilateral. To investigate the distribution of three-rooted deciduous molars in terms of symmetry, the chi-square test was used. The results showed that there was no statistically significant difference based on symmetry between groups (*p*=0.832; Table 3).

**Figure 1:**
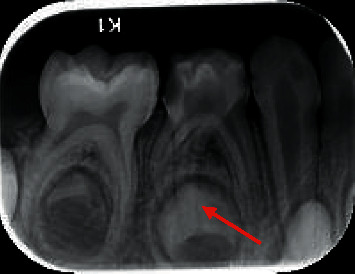
Intraoral periapical radiograph showing third root in right mandibular first primary molar.

**Figure 2:**
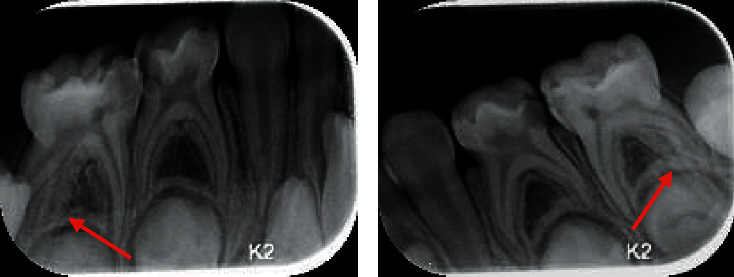
Bilateral occurrence of extra roots in second primary molars of the mandible.

Figure 1 depicts the presence of third root in right mandibular primary molar, and Figure 2 shows the bilateral occurrence of third root in second primary molar.

**Figure 3:**
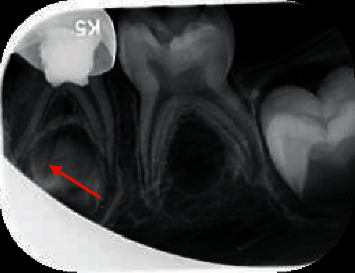
Periapical radiograph showing third root in left mandibular first primary molar.

Figure 3 shows a first primary mandibular molar after pulpotomy and stainless steel crown placement which has a distinct extra root.

## 4. Discussion

Hertwig's epithelial root sheath (HERS) is formed by the basal layer of the epithelial dental organ which consists of inner and outer enamel epithelium. Continuous elongation occurring in the HERS leads to the root growth, and the size, shape, and number of radicular pulp canals are determined in advance. Any failure or disruption during invagination of Hertwig's epithelial root sheet may result in root malformation [24].

This study was designed and carried out to investigate the prevalence of three-rooted mandibular primary molars in northwestern Iran and to assess the prevalence of this extra root in terms of sex, type of primary molar, side of the mandible, and symmetry. Based on the evidence collected, this phenomenon had a significant prevalence in our study population, and discussing it in order to administer optimal treatment plan seems to be beneficial. To the best of our knowledge, no comprehensive and detailed study in this field has been conducted in Iran.

Out of 300 patients being statistically analyzed in our study, 28 had three-rooted primary mandibular molars and the prevalence of extra root was calculated to be 9.33%. The prevalence of mandibular three-rooted deciduous molars among the total number of examined teeth was 2.5%. Of these, 16 cases (57.1%) were related to first and 12 cases (42.8%) were related to the second primary molars.

Tu et al. considered the prevalence of extra roots in only mandibular first primary molars, and it was reported to be 4.93% [17]. In a similar study conducted by Nagaveni et al., only mandibular second primary molars were examined and the total occurrence of third root among the study participants was 6.5% [25]. In another study by Srivathsa, the overall prevalence was 5.6%, of which 71.4% was related to the first primary molars and 28.6% was related to the second primary molars [16].

The overall prevalence of third root and the prevalence in first and second primary molars separately were 19.05%, 9.7%, and 27.8%, respectively, according to Song et al. [26]. In Amiri and Heidari's study, the total prevalence among primary teeth was 7.23%, of which 14.3% were mandibular first molars, 51% were mandibular second molars, and 34.7% were both mandibular first and second molars [27].

The reason for the difference in the results of the mentioned studies with the present study can be attributed to the exclusion of the first and second primary molars from the target samples in the study of Nagaveni et al. [25] and Tu et al. [17], respectively, as well as racial diversities between Taiwanese [17], Indian [16, 25], Korean [26], and southeast Iranian population [27].

Given that the imaging technique used in the study of Tu et al. [17] is different from the present and other mentioned studies [16, 25–27], vertical bitewing versus periapical radiograph, the quality of the radiographs studied, the imaging modalities, and the differences in the diagnostic opinions of the experts can be determining factors in this regard.

Multiple variables, including sample size, research design, analysis method, and studied populations, can influence the incidence of three-rooted mandibular molars. Two main approaches are used to assess the incidence of extra roots in previous research studies: (1) Radiography, which is a noninvasive method, and (2) Examination of extracted teeth which provides a more detailed knowledge of the root morphology but is considered as invasive [19].

In our study, we used periapical radiographs, instead of extracted teeth, for analyzing larger number of subjects. Extraction and CT, on the other hand, are more precise approaches for assessing the morphological root anomalies [19, 28].

Some research has been recently conducted using CT, CBCT, and micro-CT to study the prevalence of extra roots in mandibular deciduous and permanent molars. Spiral CT can show accurate three-dimensional images, which can be helpful for endodontic applications and morphologic studies, while traditional radiographs only provide a two-dimensional view of the teeth [19, 23, 28–30].

Yang et al. reported a prevalence of 27.52% among second primary mandibular molars in a group of Chinese children using cone beam computed tomography (CBCT). This is the highest percent reported to date which can be attributed to the analogy between Chinese and East Asian populations and also the contiguity of this vast country with Mongolia [31].

Although a notable number of studies have reported a higher prevalence of additional roots at the right side of the mandible, in the present study, this predilection was interrelated with the type of the primary molar (*p*=0.021), that is to say, additional roots of first primary molars tended to occur at the left side of the mandible, whereas three-rooted second primary molars have a tendency toward the right side of the mandible [17, 23, 26, 28, 29].

During the endodontic treatment, the access cavity outline form must be changed to look for extra roots or root canals in situations where they are suspected during the diagnostic stage. Clinical examination of the tooth crown and investigation of the cervical morphology of the roots using periodontal probing can help identify an extra root in addition to a radiographic diagnosis. In conjunction with a cervical prominence or convexity, an extra cusp (tuberculum paramolare) or more pronounced occlusal distal or distolingual lobe might suggest the existence of an additional root. The extra root or canal's orifice is located disto- to mesiolingually from the distal root's main canal(s). A trapezoidal shape is formed when the triangular cavity is extended to the distolingual side [32].

Some recent studies have been conducted focusing on the association between the simultaneous occurrence of supernumerary roots in primary and permanent molars. Research shows that the deciduous mandibular second molars and permanent mandibular first molars belong to the same molar field because of their same time of development and similar crown morphology. This theory was first proposed by Butler [33–35]. In a study published by Song et al. to evaluate this association, the probability of seeing extra roots in both second primary and first permanent mandibular molars was found to be 94.3% [26].

## 5. Conclusion

Based on the data obtained, a relatively high prevalence of extra root was recorded in children living in northwestern Iran. However, in pediatric dentistry references and practical training of dental students, less attention is paid to this issue and its details. Meticulous interpretation of radiographic images along with considering racial and geographical characteristics would improve diagnosis and treatment.

Addressing this issue and its correlation with variations in both primary and permanent dentitions in future studies seems to be advantageous.

## Figures and Tables

**Table 1:** Comparison of the frequency distribution of the type of three-rooted deciduous teeth in terms of sex.

	Gender		Type of the molar			Total
			None	D	E	
*Gender*	Girls	*N*#	150	10	5	165
		Percent	55.1%	62.5%	41.7%	55%
	Boys	*N*#	122	6	7	135
		Percent	44.9%	37.5%	58.3%	45%

*χ* ^2^_(2)_ = 1.23, *p*=0.541						

**Table 2:** Comparison of the frequency distribution of the type of three-rooted deciduous teeth in terms of side of the mandible.

Side of the mandible			Type of the molar		Total
			D	E	
Side of the mandible	Left	*N*#	11	2	13
		Percent	68.8%	16.7%	46.4%
	Right	*N*#	4	9	13
		Percent	25%	75%	46.4%
	Both	N#	1	1	2
		Percent	6.2%	8.3%	7.2%

*χ* ^2^_(2)_ = 7.74%, *p*=0.021					

**Table 3:** Comparison of the frequency distribution of the three-rooted deciduous teeth in terms of symmetry.

Symmetry			Type of the molar		Total
			D	E	
Symmetry	Unilateral	*N*#	15	11	26
		Percent	93.8%	91.7%	92.9%
	Bilateral	*N*#	1	1	2
		Percent	6.2%	8.3%	7.1%

*χ* ^2^_(1)_ = 0.45, *p*=0.832					

## Data Availability

The data supporting the results discussed in this article are available upon request from the corresponding author.
